# Bibliometric analysis and visualization of lipid droplets in the central nervous system: research hotspots and Frontiers (2000–2024)

**DOI:** 10.3389/fnagi.2025.1534368

**Published:** 2025-03-13

**Authors:** Yanan Wang, Simin Chen, Xinyi Lv, Jiahui He, Xiao Liang, Yuehan Song

**Affiliations:** ^1^College of Traditional Chinese Medicine, Beijing University of Chinese Medicine, Beijing, China; ^2^Department of Stomatology, Qianfoshan Hospital in Shandong Province, Jinan, China

**Keywords:** bibliometrics, visual analysis, lipid droplets, nervous system, hotspots

## Abstract

**Objective:**

The aim of this study is to conduct bibliometric analysis and visualization of the research progress of lipid droplets in the central nervous system in detail using CiteSpace, VOSviewer, and to explore the current research status, hotspots, and research trends, with a view to providing a basis for future research.

**Methods:**

This study utilized the Web of Science database to search for 1,066 relevant publications on lipid droplets in the central nervous system from 2000 to 2024. Bibliometric analysis was conducted using CiteSpace and VOSviewer software, producing metrics such as annual publication trends, contributions by countries, institutions, and authors, keyword co-occurrences, and reference co-citation networks. The literature of 25 years or so was explored visually to identify the important areas of lipid droplets in neurological research.

**Results:**

Miguel Lopez is the largest contributor to the relevant literature with 10 publications. The United States, China, Johns Hopkins University, the University of Cambridge, and Zhejiang University are the top contributors in terms of publication volume in this research area. Current research emphasizes the mechanisms of lipid droplets in oxidative stress, neuroinflammation, and related degenerative diseases, with a particular focus on Alzheimer's Disease.

**Conclusion:**

Our analysis suggests enhancing collaboration among countries, institutions, and authors in clinical and basic research on brain lipid droplets.

## 1 Background

### 1.1 Introduction

Lipid droplets, originating in the intracellular endoplasmic reticulum, consist of a core of neutral lipids like triglycerides and cholesteryl esters. They play a role in cellular lipid turnover and stress responses in the central nervous system by managing the storage of surplus fatty acids, cholesterol, and ceramides (Smolič et al., 2021a). Lipid droplets, typically scarce in the central nervous system (Etschmaier et al., 2011), show altered accumulation in aging (Garcia-Cazorla and Saudubray, 2018) and various neurological disorders such as Alzheimer's (Haney et al., 2024), Huntington's (Hamilton et al., 2015), multiple sclerosis (Aditi et al., 2016), including neurodegenerative disorders, strokes, and gliomas (Teixeira et al., 2021; Tracey et al., 2018; Farmer et al., 2019), and are predominantly appeared in glial cells that provide nutritional, metabolic, and immune support to neuronal networks. Lipid droplet accumulation is implicated in the development of neurological diseases, with its metabolic disruption potentially leading to central nervous system disorders (Zhang et al., 2025).

Research in Cell demonstrated that in a Drosophila model, lipid droplet accumulation in glia precedes or triggers neurodegeneration. Reducing this accumulation and lipid peroxidation significantly delays neurodegeneration onset (Liu et al., 2015). A mouse model exhibiting anxiety-like behavior due to a high-fat diet showed a notable rise in lipid droplets within hippocampal microglia, a crucial brain area for emotion and stress regulation (Yao et al., 2024).

Recent advancements in brain lipid droplet research have resulted in a growing number of related publications. Nonetheless, there is a lack of literature that comprehensively explores, analyzes, and summarizes progress in this area. Bibliometrics is an important discipline dedicated to assessing the quantitative characteristics, trends, and scholarly impact of scientific literatures (Zhou et al., 2022; Liao et al., 2023). Researchers can use search systems and applications to thoroughly assess the quantity and quality of publications, as well as to explore research advances and trends in the field.

### 1.2 Literature review

Existing literatures lack a systematic compendium of lipid droplet research in the CNS, especially parsing across diseases and technological evolution, which is the innovative foothold of this study. Although no bibliometric studies have been conducted in the field of lipid droplet research, some scholars have attempted to summarize the progress of lipid metabolism and neurodegenerative diseases through bibliometric methods. Jallow et al. (2024) concluded that acrolein, as both an environmental pollutant and an endogenous compound, has become a hot topic of research by modulating the role of β-amyloid in Alzheimer's disease. There is also a study to visualize and analyze lipid autophagy (autophagic degradation of lipid droplets) between 2013 and 2023, and the hotspot diseases include neurodegenerative diseases (Alzheimer's disease, Parkinson's disease), which has a certain significance on the mechanism of lipid droplet metabolism disorders that are the concern of the present study, and suggests that it is necessary to pay attention to the integration analysis of the dynamic equilibrium of lipid droplets with the aberrant protein clustering, lipid metabolism, and organelle interactions in the neurodegenerative pathology. This suggests the need to focus on the integration of lipid droplet dynamic balance with abnormal protein aggregation, lipid metabolism and organelle interactions in neurodegenerative pathologies (Zhao et al., 2024). Although the bibliometric study of iron death in Parkinson's disease did not focus directly on lipid droplets, its methodological framework (e.g., co-occurrence network analysis, synonym detection) and the similar or cross-cutting themes revealed (oxidative stress, phospholipid oxidation accumulation, mitochondrial dysfunction) provide important references to the bibliometric analyses of lipid droplet studies, suggesting that imbalances in lipid homeostasis may be involved in neurological pathology through multiple pathways (Lu et al., 2023).

This study conducted a comprehensive analysis of literature from the past two decades on Web of Science, examining national and institutional publication sources, author co-authorship, reference co-citation, and keyword co-occurrences to elucidate trends, identify research priorities in lipid droplets, and predict future research directions (Li et al., 2022).

## 2 Materials and methods

### 2.1 Data sources and search strategies

**Figure 1:**
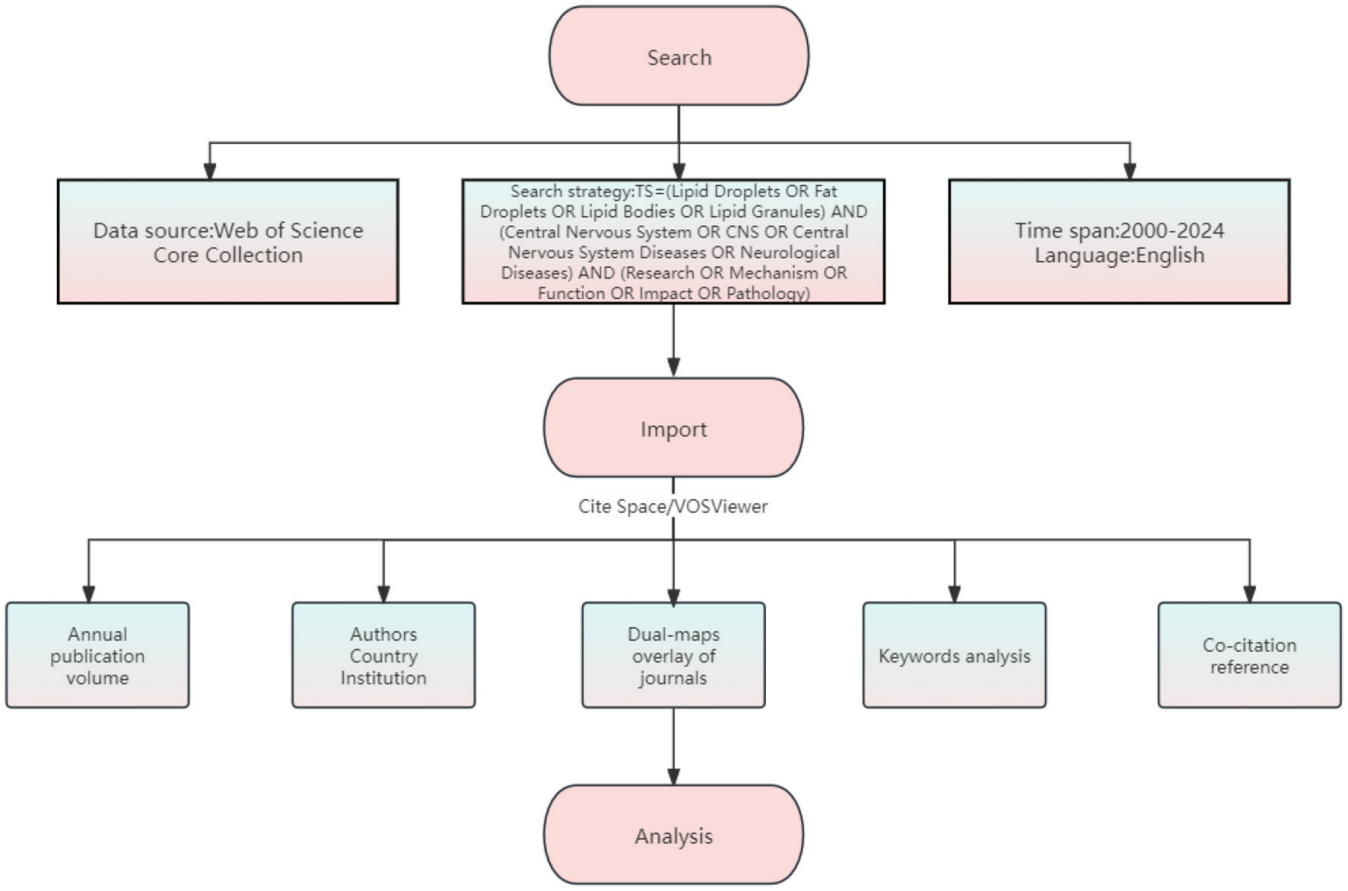
Flowchart for research.

The WOS database has over 12,000 influential journals and is widely regarded as the most complete and trustworthy bibliometric analysis database (Lyu et al., 2022). As shown in Figure 1, studies from 2000 to 2024 were searched using the WOS Core Collection. The following terms were searched in the subject: (Lipid Droplets OR Fat Droplets OR Lipid Bodies OR Lipid Granules) AND (Central Nervous System OR CNS OR Central Nervous System Diseases OR Neurological Diseases) AND (Research OR Mechanism OR Function OR Impact OR Pathology). The selection was restricted to English-language original articles and reviews. A total of 1,066 articles that met the criteria were ultimately included.

### 2.2 Visualization analysis tool

A total of 1,066 documents in plain text format were imported into CiteSpace and VOSviewer for bibliometric analysis and visualization (van Eck and Waltman, 2010; Chen, 2004). The analysis encompassed publication volume over time, authorship patterns, national and institutional co-authorships, keyword co-occurrence, clustering, and bursts. The time zone selected for data processing was from 2000 to 2024, with a time period of “1”; term sources included “title”, “abstract”, The term sources include “title”, “abstract”, “author” and “keywords”, etc. The node types include author, organization, keywords, etc. The rest of the settings are set as default.

## 3 Results

### 3.1 Annual trend of publication volume

**Figure 2:**
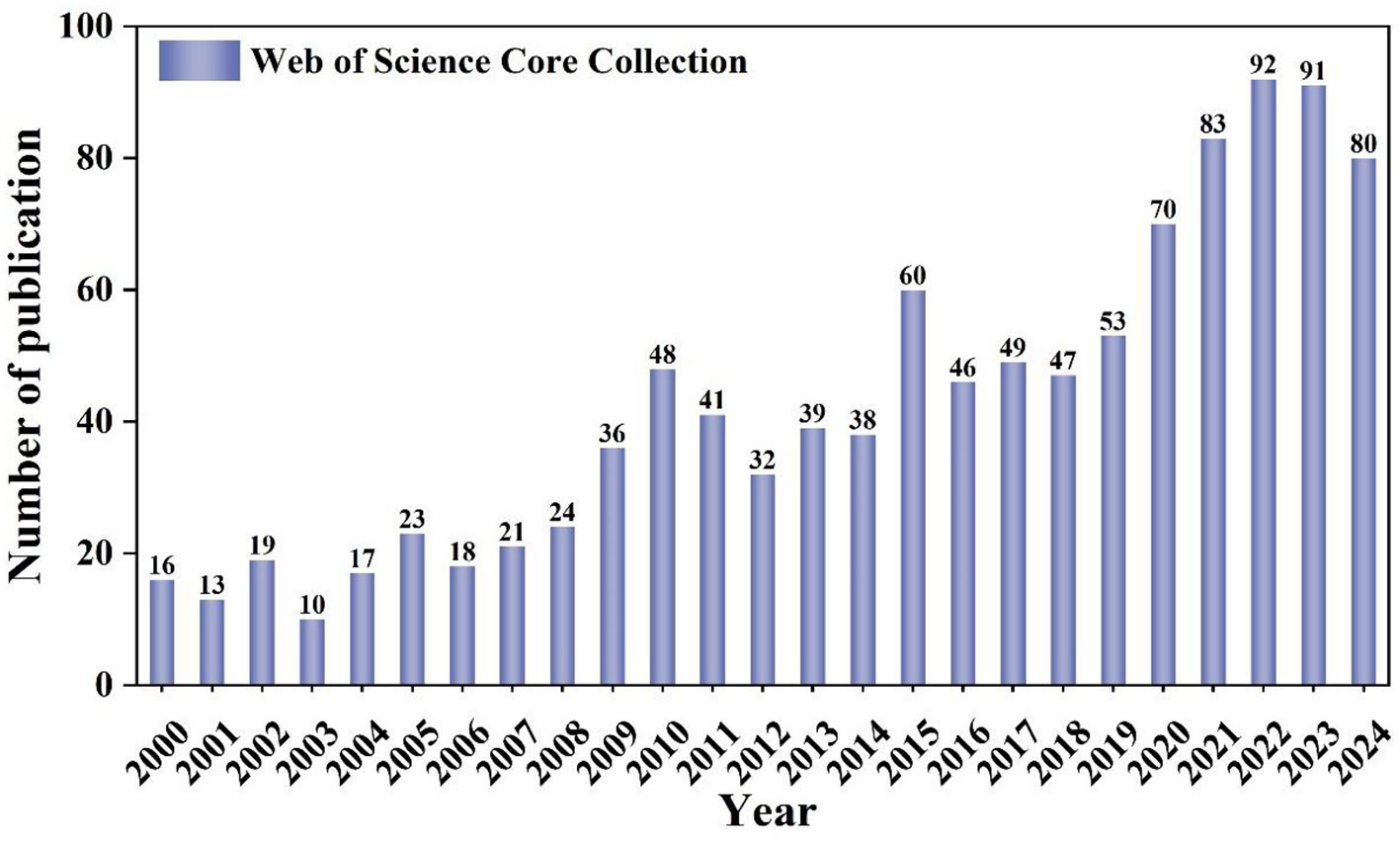
Annual publication quantity and trend of research on lipid droplets in neurological diseases.

Figure 2 illustrates a general and continuous upward trend in the amount of literature concerning lipid droplets in the nervous system over the past two decades. This trend can be categorized into three distinct phases: the initial phase (2000–2010), when the field was in its infancy and the level of research was low. However, as time went on and advancements in understanding neurodegenerative disease mechanisms, such as Alzheimer's and Parkinson's, along with technological progress, made relevant experimental observations more feasible (Cole et al., 2002; Outeiro and Lindquist, 2003), lipid droplets began to be emphasized as an important component of cellular energy and lipid metabolism, and the literature increased year by year. This was followed by the second phase (2010–2015), marked by fluctuations in the volume of publications. 2016–2024 belongs to the third phase, the research on lipid droplets and their role in neurological diseases is still growing steadily, showing the scientific value and research potential of the relationship between lipid droplets and the CNS, and the formation of LDs is intricately linked to pathological aspects such as mitochondrial dysfunction, neuroinflammation, aging, neurodegeneration, and *ApoE* risk genes.

### 3.2 Authors' mapping analysis

**Figure 3:**
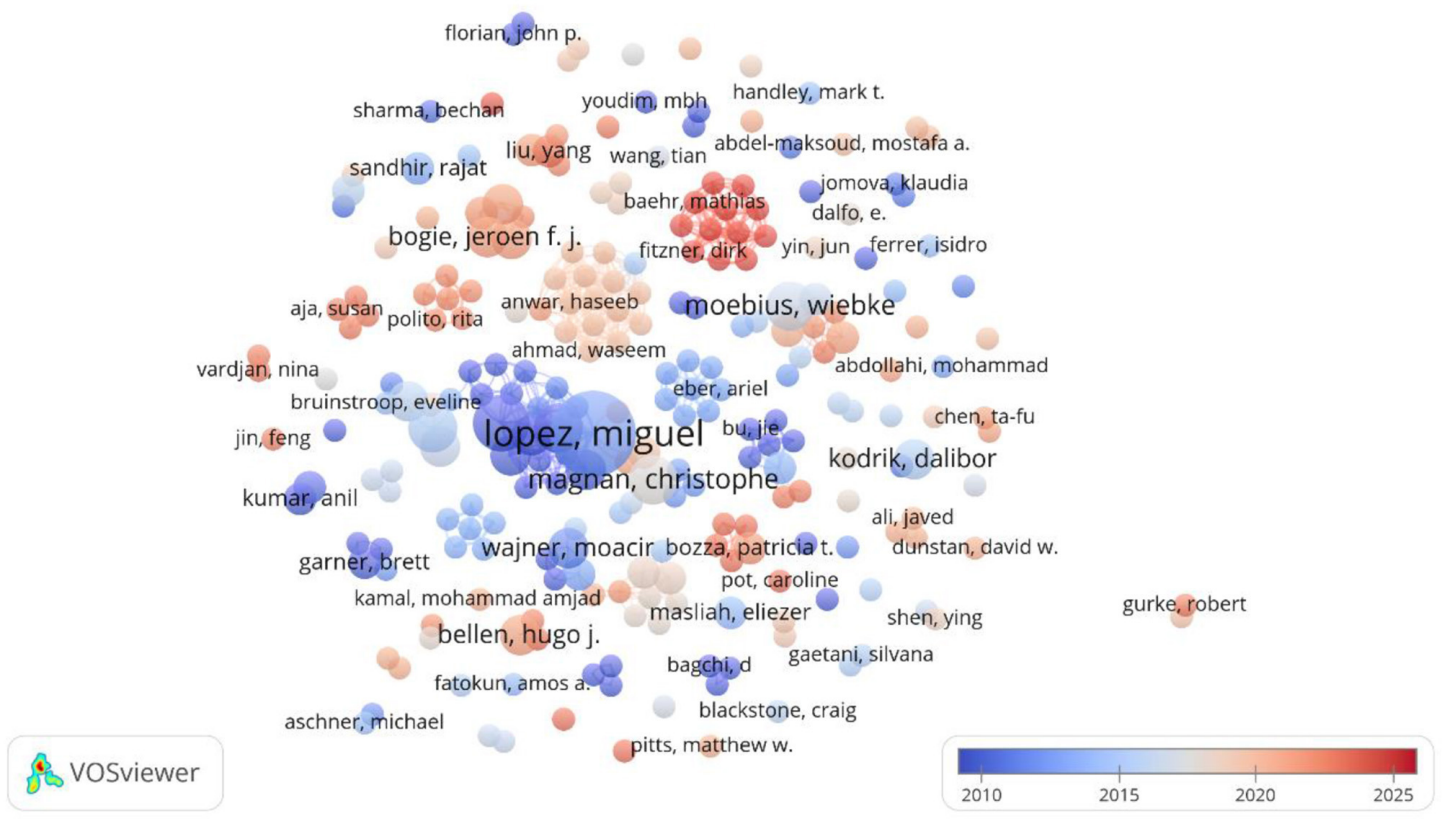
Co-occurrence of principal investigators of lipid droplets and their role in neurological disorders.

The larger the circle of a word in the VOSviewer graph is the more central the node, and the same color represents a closer relationship that will form similar themes; the lower right corner indicates the average time of occurrence of the word, and the darker the color, the earlier the occurrence (Wei et al., 2022). As shown in Figure 3, the highest contributing author was Miguel Lopez, with 10 publications, becoming a major early mover in the field, not only deepening the understanding of the function of lipid droplets, but also shedding light on their role in the study of metabolic and neurodegenerative disorders in particular. Ruben Nogueiras, with 6 publications, explored lipid droplets' impact on energy metabolism and neurological health, offering insights into the link between obesity and central nervous system disorders.

In addition, Carlos Dieguez, Robert H. Eckel, Christophe Magnan, and Wiebke Moebius, all with 5 publications, have focused on the cellular signaling and metabolism of lipid droplets as well as their interactions with neuronal cells.

### 3.3 Analysis of countries and institutions

**Figure 4:**
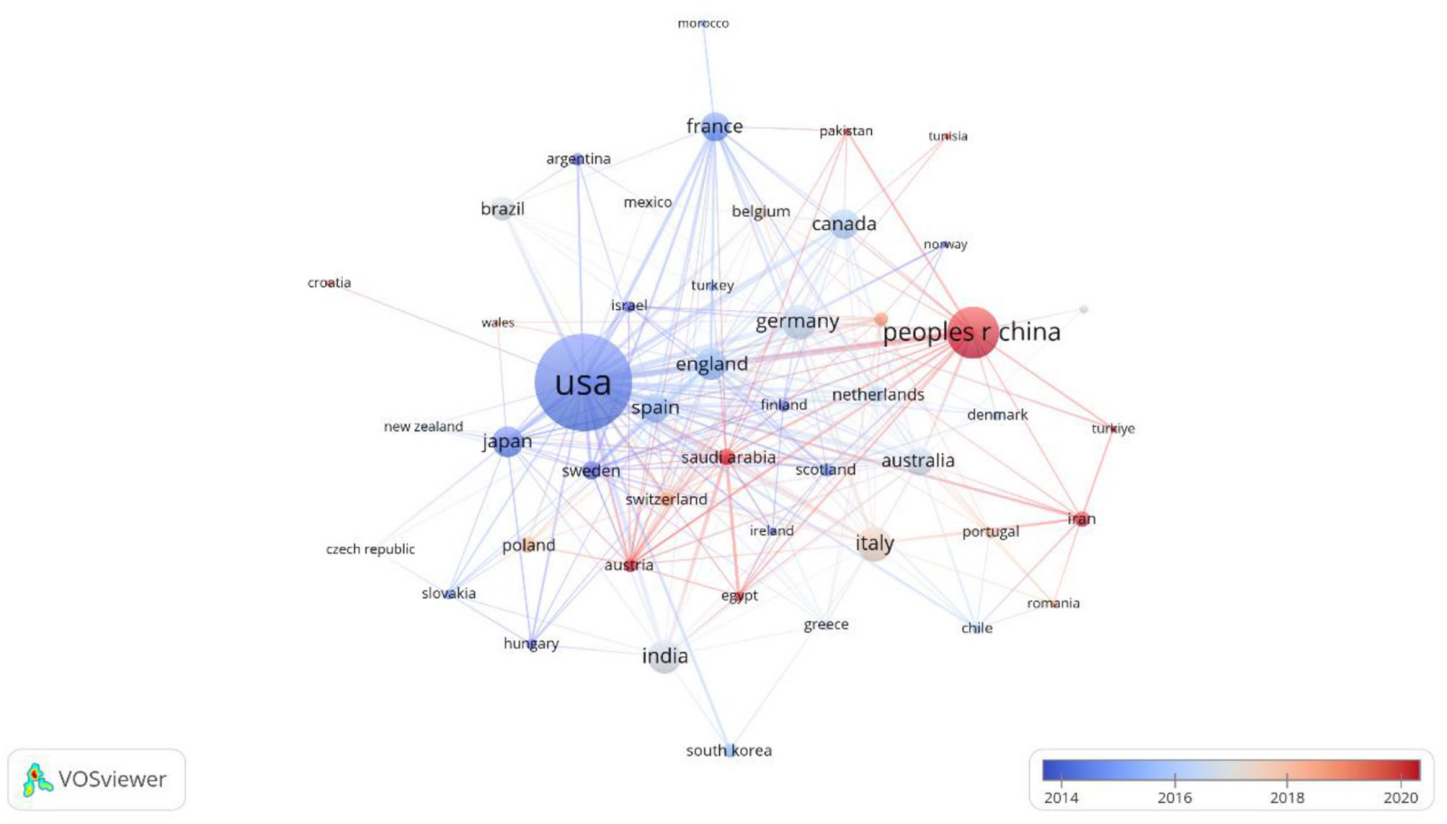
National network for the study of lipid droplets in central nervous system diseases.

**Figure 5:**
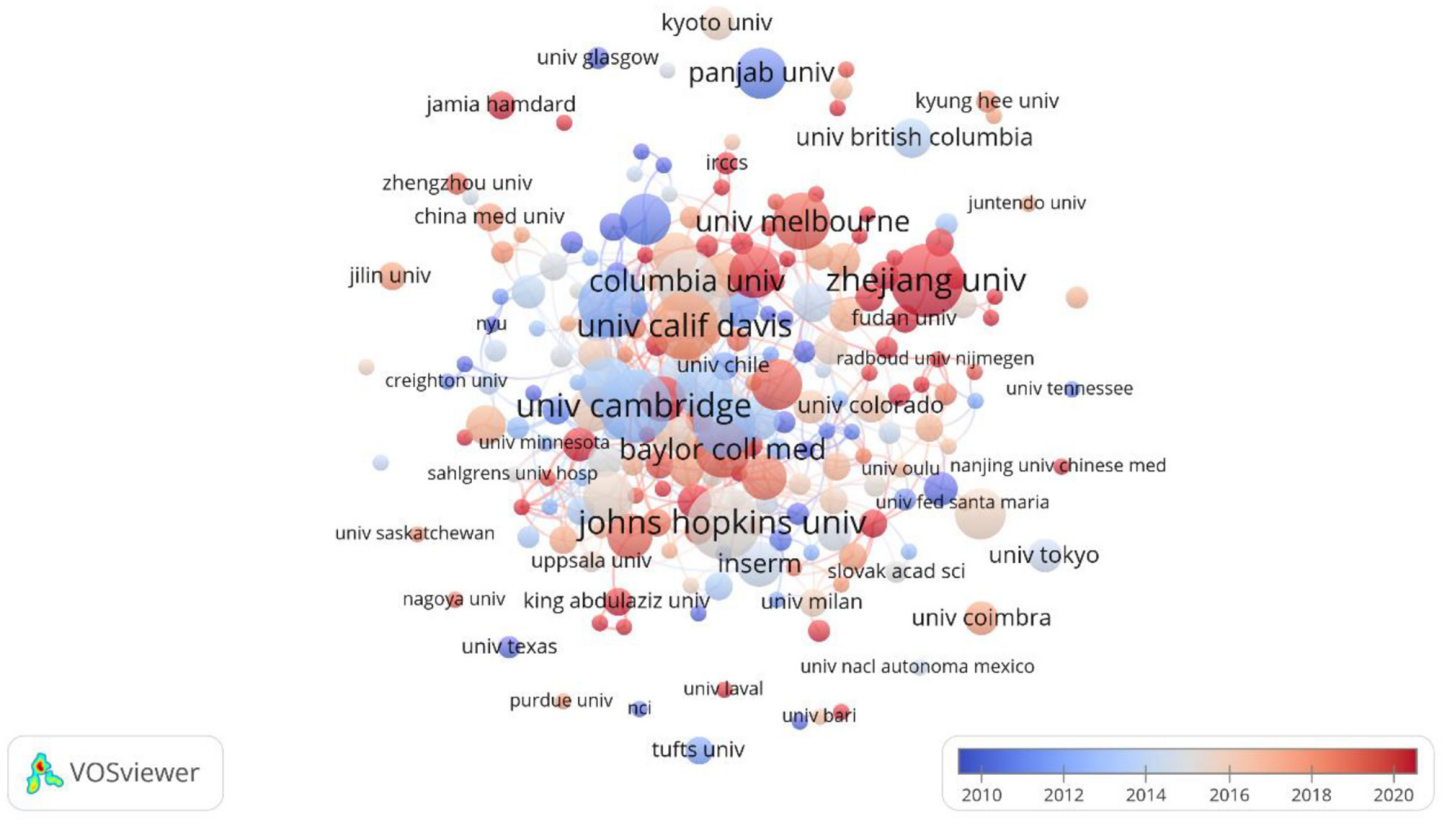
Institutional map of the study of lipid droplets in central nervous system disorders.

Figures 4, 5 present a network map of countries/institutions where lipid droplets have been studied in the field of the nervous system. Circles denote various countries and institutions, with their size reflecting the volume of published articles. The width of the links shows the intensity of the collaboration, while clusters in various colors denote countries and institutions with robust partnerships (Li J. et al., 2024). A total of 47 countries and 220 institutions have published articles globally, reflecting the research contributions and focus of each country in this area. The leading countries are the United States, China, Germany, Italy, and India. Among them, the U.S., as the leader of the research, has collaborated most closely with other countries and published 378 related papers, which is among the pioneers in the field.

In addition, the output institutions are mostly prestigious research universities, most of which have close cooperation with each other in the field of lipid droplets, but there are still some institutions that do not have any international exchange and cooperation behaviors. The top 10 institutions are Johns Hopkins University, University of Cambridge, Zhejiang University, University of California, Davis, University of Santiago de Compostela, Columbia University, and Harvard University. Compostela, Columbia University, and Harvard University, which were cited an average of 889 times per article and up to 1,743 times.

### 3.4 Journal analysis

**Figure 6:**
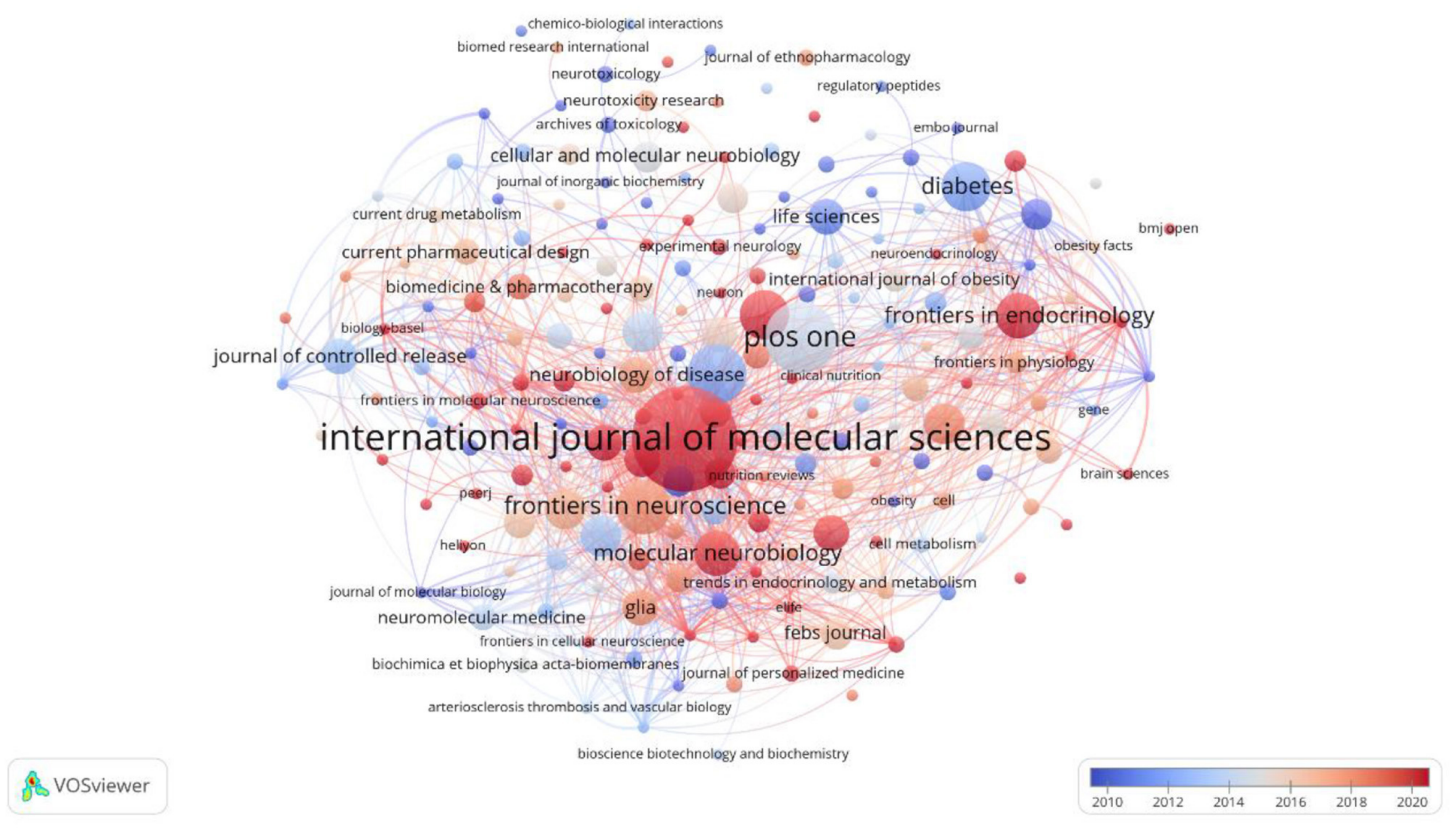
Map of journal publications on lipid droplet research in central nervous system disorders.

Among the 201 journals, *International Journal of Molecular Sciences* ranks first in publishing 23 related studies and co-authored works, with 668 citations. Papers in this journal often employ a variety of experimental techniques, such as molecular and cellular biology, and are dedicated to exploring the structure and function of lipid droplets, especially in the regulation of metabolism in nerve cells. Secondly, numerous high-impact Q1 journals are available, including *Cell* (IF = 64.5), *Journal of Controlled Release* (IF = 10.5), *Proceedings of the National Academy of Sciences of the United States of America* (IF = 11.1), and other high-impact journals in the Q1 region have similarly published a range of influential papers. Refer to Figure 6, which shows that both node and font size increase with the number of articles a journal publishes, while the thickness of the connecting lines represents collaboration density.

### 3.5 Examination of key research areas

#### 3.5.1 Keyword co-occurrence analysis

**Figure 7:**
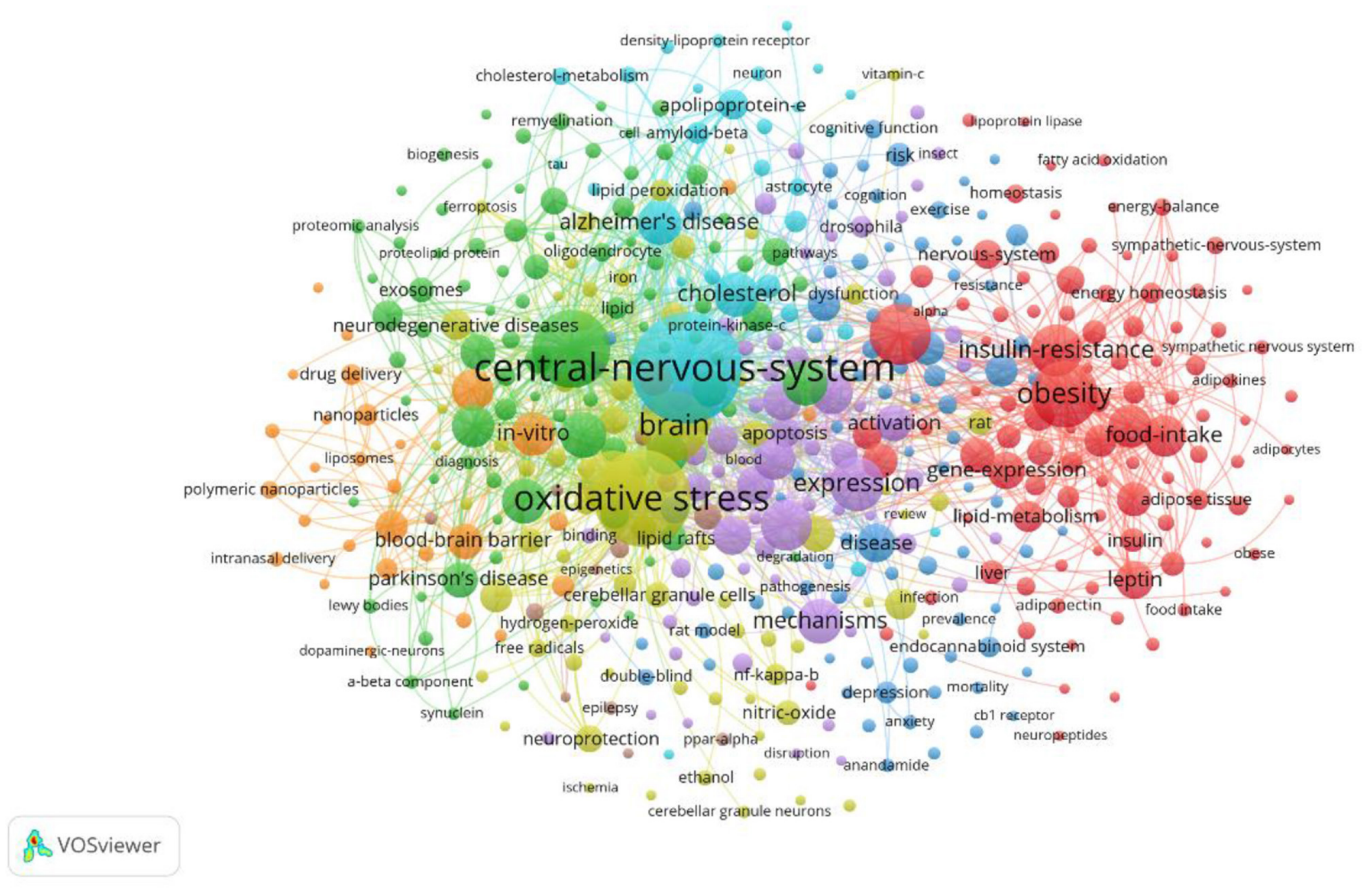
Keyword co-occurrence network for the study of lipid droplets in central nervous system disorders.

The co-occurrence of high-frequency words reflects the research hotspots. As shown in Figure 7, the relationship network of concurrent keywords is shown to visualize the frequency of occurrence of the main keywords in the research field (refer to Supplementary material). In addition to the central theme word, the most frequently occurring keyword is “Oxidative Stress” (frequency: 181), which is found to be one of the key mechanisms of lipid droplet metabolism disorders, and it impacts neuronal survival and contributes to neurodegenerative diseases like “Alzheimer's Disease” (frequency: 161), “Parkinson's Disease” (frequency: 69). Additionally, “Lipid Peroxidation” (frequency: 98) is frequently associated with oxidative stress. The co-occurrence of these keywords highlights the complex interrelationships between oxidative stress, lipid droplets and neurodegenerative diseases in lipid droplet studies in the CNS (Mani et al., 2023).

On the other hand, states such as “Obesity” (frequency: 30) and “Metabolic Syndrome” (frequency: 21) may exacerbate the progression of neurodegenerative diseases through lipid droplet mechanisms. Studies have shown that different “Food Intake” (frequency: 44), especially the “High Fat Diet” (frequency: 8), may affect the progression of neurodegenerative diseases by influencing “Body Weight” (Frequency: 49), “Insulin Resistance” (Frequency: 72), cellular “Metabolism” (Frequency: 44), and neurological “Inflammation” (Frequency: 3). “Inflammation” (frequency: 18) contributes to lipid droplet accumulation in nerve cells, thereby accelerating neurodegenerative pathologies. The co-occurrence of these keywords provides new perspectives for understanding the potential mechanisms of lipid droplets in CNS diseases.

#### 3.5.2 Analysis of keyword clustering

**Figure 8:**
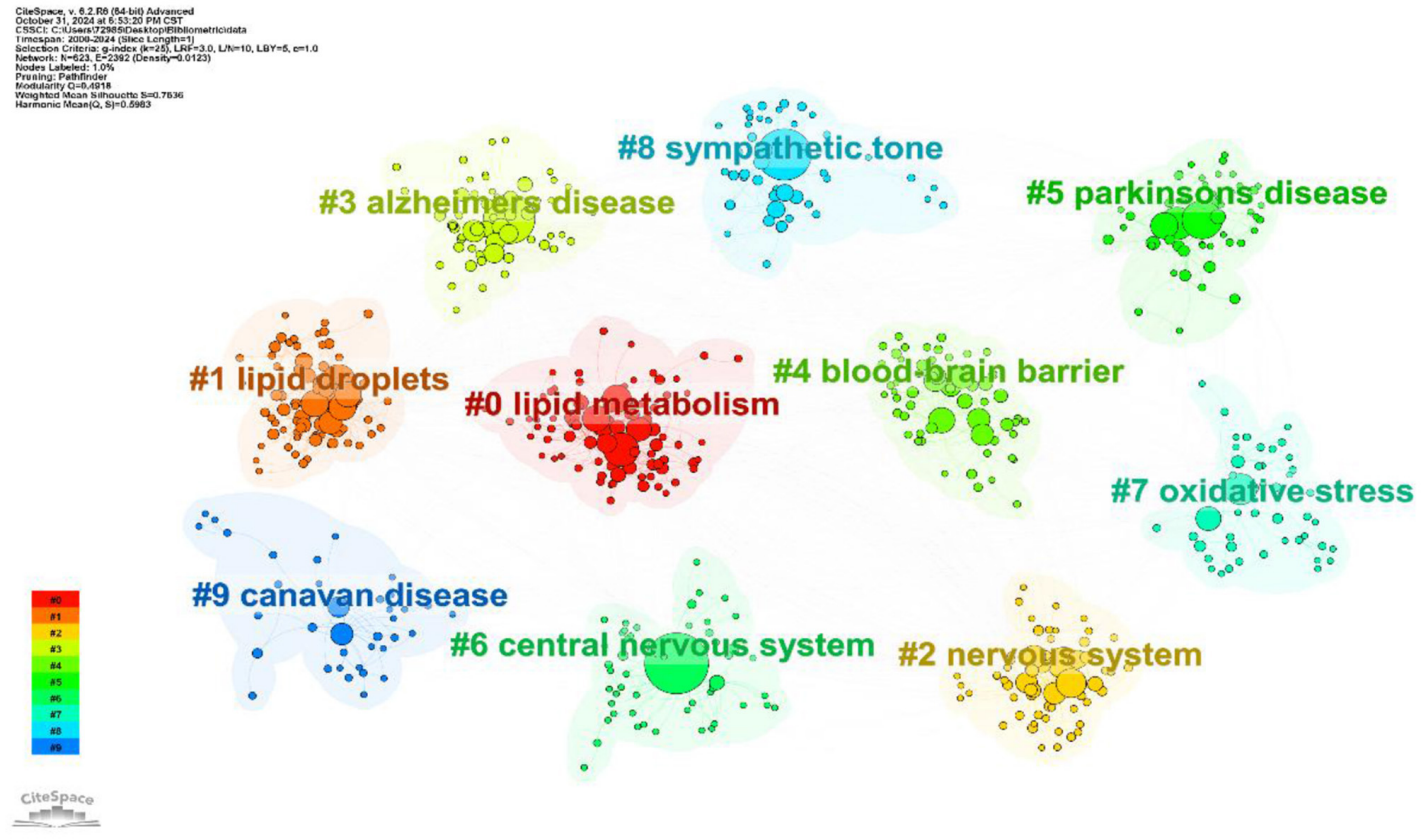
Keyword clustering diagram of lipid droplets in central nervous system diseases.

In keyword clustering analysis, Silhouette Score is an important quantitative index to assess the quality of cluster analysis. Compared with the traditional subjective classification methods, it can effectively avoid the cognitive bias of researchers in the subject classification, especially when dealing with large-scale literature keywords, it can accurately identify the optimal number of clusters and verify the applicability of the algorithm. The numerical interval is between −1 and 1, the larger the value, the better the clustering effect. It is usually >0.5 to indicate that the clustering effect is a reasonable criterion, and >0.7 is considered to reach a convincing degree. As shown in Table 1 and Figure 8, the keyword clustering of the study was mainly related to #0 lipid metabolism, #1 lipid droplets, #2 nervous system, #3 Alzheimer's disease, #4 blood-brain barrier, #5 Parkinson's disease, #6 central nervous system, #7 oxidative stress, #8 sympathetic tone, #9 canavan disease. central nervous system, #7 oxidative stress, #8 sympathetic tone, #9 canavan disease. These labels are further categorized: #0, #1, #2, and #6 constitute the first category, which focuses primarily on the research topic; #4, #7, and #8 constitute the second category, which centers on exploring the mechanisms of lipid droplets; and #3, #5, and #9 comprise the third category, which examines lipid droplet-related diseases.

**Table 1:** Keyword clustering analysis.

**Silhouette score**	**Year**	**Keywords**
0.736	2011	Lipid metabolism; adipose tissue; insulin resistance; nervous system; food intake|fatty acid oxidation; fatty acids; Alzheimer's disease; sexual dimorphism
0.744	2014	Lipid droplets; lipid metabolism; chorea acanthocytosis; axonopathy; autophagy|lipid droplet; droplet; solid lipid nanoparticles; neuron;
0.682	2013	Lipid metabolism; nervous system; insulin resistance; receptor; food intake|adipose tissue; sympathetic nervous system; neuropeptidey; pancreatic beta cells
0.767	2010	Alzheimer's disease; app processing; Parkinson's disease; gaucher disease; lysosomal storage disease|neurodegenerative diseases; coronary artery disease; sex difference; brain disorder
0.751	2015	Blood-brain barrier; extracellular vesicle; neurovascular unit; ischemic stroke; therapeutic measures|drug delivery; tight junction modulator; nasal drug delivery; absorption enhancer
0.806	2007	Parkinson's disease; pore formation; molecular simulations; lewy bodies; biological markers|oxidative stress; maple syrup urine disease; phosphoryl transfer network; magnetic resonance spectroscopy
0.75	2005	Central nervous system; arcuate nucleus; gene expression; pioglitazone; insulin sensitivity|Alzheimer's disease; Parkinson's disease; membrane transporters; lipids
0.774	2008	Oxidative stress; yttrium oxide nanoparticles; programmed cell death; e suppresse; sesbania grandiflora|brain; model; mechanism; stress
0.794	2011	Oxidative stress; sympathetic tone; blood pressure; kainic acid; domoic acid|lipid peroxidation; neurodegenerative diseases; glutamatergic synapses; heavy metals
0.797	2008	Canavan disease; oxidative stress; n-acetylaspartic acid; twitcher mouse; lipids|acid amide; cannabinoid cb2; 2 arachidonoyl; therapeutic target

#### 3.5.3 Keyword time trend analysis

**Figure 9:**
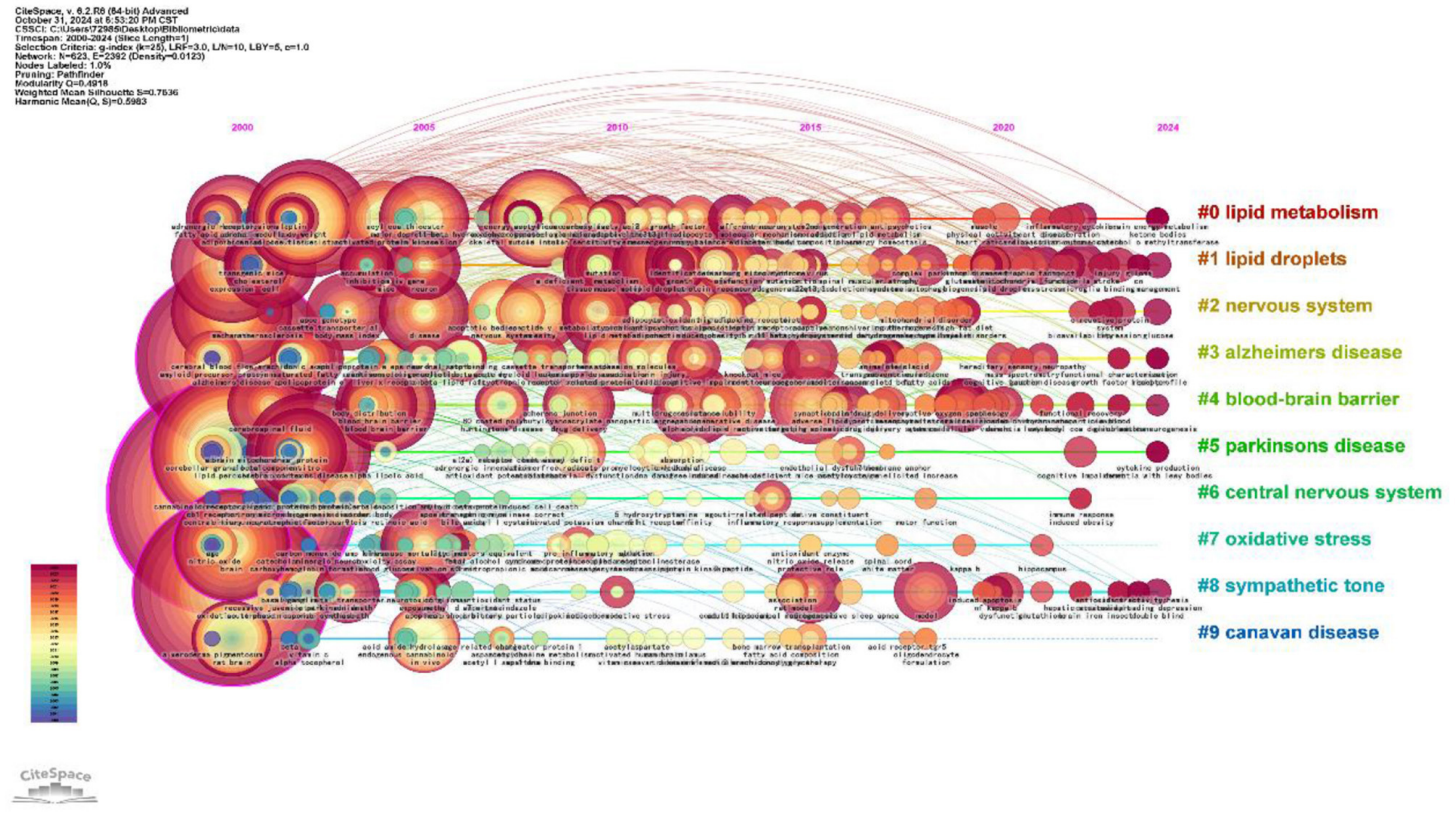
Time line diagram of keywords for the study of lipid droplets in central nervous system disorders.

Temporal trend analysis reflects the historical evolution and frontier of the field's hotspots in recent years. Each CiteSpace node represents a single item, with its size indicating importance or influence; colored circles indicate citations to a specific article, with its overall size reflecting the number of times it was cited, and its color representing the time period in which it was cited. Node centrality is represented by the outermost purple circle, where increased thickness signifies higher centrality levels. Higher centrality values signify increased prominence or influence (Liu et al., 2019). Figure 9 illustrates that each horizontal line denotes a cluster, with smaller label numbers indicating larger clusters. Node size represents co-citation frequency, while links denote co-citation relationships. The colors of the nodes and lines indicate the different years of citation. From 2000 to 2002, the keywords were concentrated in “central nervous system” (256), “oxidative stress” (181), “Alzheimer's disease” (161), and “lipid peroxidation” (98) that underlie lipid droplets and central nervous system (CNS) diseases, revealing a relationships on the association between oxidative stress and Alzheimer's disease were revealed. In addition, “insulin resistance” (72) and “Parkinson's disease” (69) also began to receive attention during this period, suggesting that research on lipid droplets in relation to insulin resistance and Parkinson's disease was gradually emerged. From 2005 to 2012, the keywords “lipid metabolism” (54), “mouse model” (47), “blood-brain barrier” (43), and “lipid metabolism” (54) were used to describe the relationship between lipid droplets and insulin resistance and Parkinson's disease (54). “mouse model” (47), “blood-brain barrier” (43), and “protein” (41) gradually became the focus of research. Researchers utilized animal models to study the impact of lipids on blood-brain barrier integrity and neuronal function. From 2013 to 2024, researchers have increasingly focused on lipid droplets' roles in energy metabolism and cell signaling (Smolič et al., 2021b; Bailey et al., 2015), with significant attention given to the keywords “neurodegenerative diseases” (13), “energy metabolism” (14), “extracellular vesicle” (13), and “inflammation” (18). In addition, the appearance of the keyword “inflammation” indicates that researchers began to recognize the importance of lipid droplets in the inflammatory response.

#### 3.5.4 Analysis of keyword bursts

**Figure 10:**
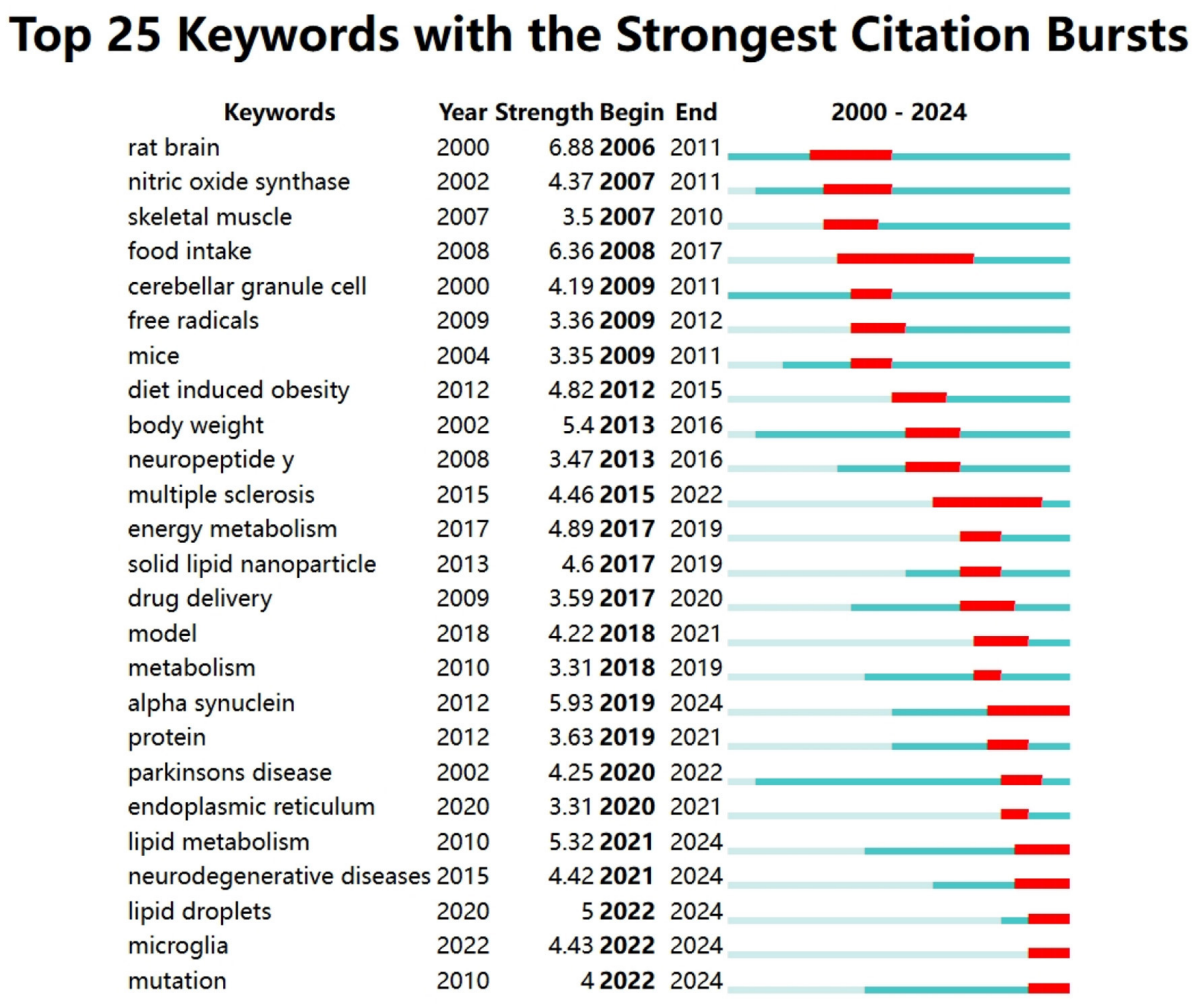
Keyword emergence map for lipid droplets in CNS disease research.

According to the results of CiteSpace analysis, from Figure 10, we can see the top keywords, importance index, start year and end year of lipid droplets in CNS disease research field. First of all, the appearance of keywords “rat brain” (burst intensity 6.88), “nitric oxide synthase” (burst intensity 4.37) marked the initial role of lipid droplets in CNS Exploration. From 2008 to 2015, “food intake” (burst intensity 6.36), “free radicals” (burst intensity 3.36), “diet induced obesity” (burst intensity 4.82), “body weight” (burst intensity 5.4), and “neuropeptide Y” (burst intensity 3.47) gradually became the most important factors in the study. This reflects the interest in the role of diet and obesity in lipid droplet metabolism and its role in neurological disorders. Between 2017 and 2024, researchers explored the roles of lipid droplets as intracellular lipid storage and metabolic regulation centers, with “energy metabolism” (burst intensity 4.89), “lipid droplets” (burst intensity 5.00), and “microglia” (burst intensity 4.43) gaining attention. The role of lipid droplets in neuroimmune responses began to be recognized, especially in the interactions between neurons and glial cells with the emergence of “microglia” (Garcia Corrales et al., 2021; Ralhan et al., 2021), and their potential impact in neuroinflammatory and neurodegenerative diseases was recognized.

#### 3.5.5 Reference highlighting

**Figure 11:**
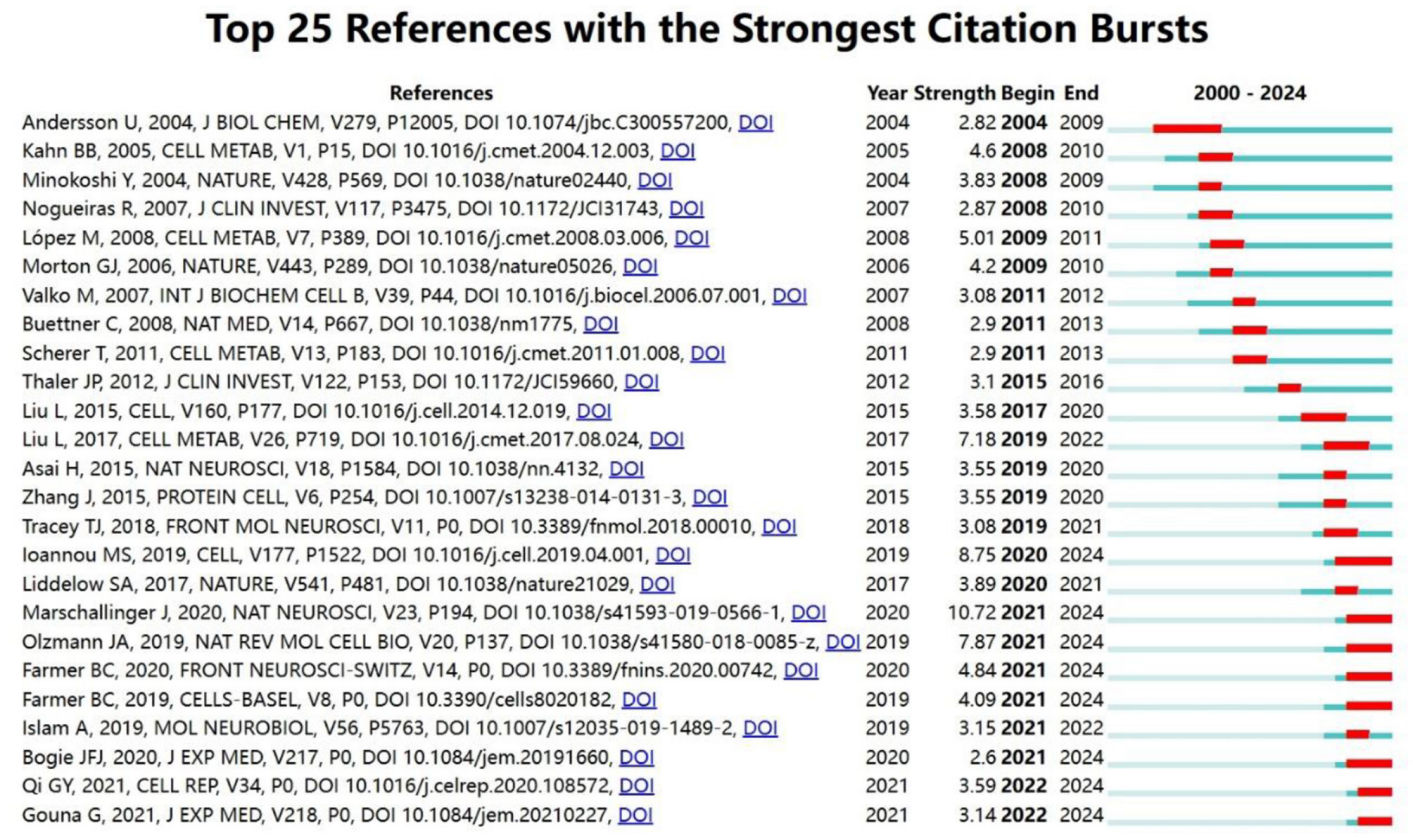
Map of hot year emergence of references for lipid droplet studies in CNS disorders.

Figure 11 illustrates the detection of hotspot year bursts in references related to lipid droplets in CNS disorders from 2000 to 2024. The blue line represents the 2000–2024 timeframe, while the red line highlights the burst periods. The top 25 most-cited references in articles from 2000 to 2024 serve as a key indicator of scholarly importance, reflecting their academic impact within the field. The blue bars indicate the publication time of the references, and the red bars indicate the emergence of citation frequency. Several articles were published in influential journals such as *Cell, Cell* subspecialty *Cell Metabolism* and *Nature* subspecialties *Nature Reviews Molecular Cell Biology, Nature Neuroscience*, and so on. The primary articles consist of 18 experimental studies and 7 review articles. Among them, the burst intensity of experimental articles published in *Nature Neuroscience* by Marschallinger J et al. in 2020 reaches 10.72, bursting from 2021 to 2024. This study investigated lipid droplet accumulation in microglia within aging brains and its link to neurodegenerative diseases. It was found that both mouse and human brain microglia significantly accumulate lipid droplets, becoming “lipid droplet-accumulating microglia”, which exhibit impaired phagocytosis, elevated ROS production, and increased secretion of pro-inflammatory cytokines (Marschallinger et al., 2020). Next is the review published in *Nature Reviews Molecular Cell Biology* by Olzmann JA et al. in 2019, which outlined the dynamic changes of lipid droplets and their functions within cells (Olzmann and Carvalho, 2019), and Liu et al.'s (2017) article published in *Cell Metabolism*, with burst intensities of 7.87 and 7.18, respectively. Two articles with high citation rates and far-reaching impact by Liu et al. respectively, are an experimental study published in *Cell* in 2015 by Liu et al. from Baylor College of Medicine in the U.S. The outbreaks occurred from 2017 to 2020. The study observed that mitochondrial dysfunction in neurons increases reactive oxygen species (ROS) levels, subsequently activating c-Jun-N-terminal kinase (JNK) and SREBP. These factors enhance lipid synthesis in neurons and lipid droplet formation in glial cells. These lipid droplets further contribute to the development of neurodegenerative diseases by increasing lipid peroxidation levels. Research indicates that lipid droplet formation is a transient event preceding neurodegeneration in Drosophila and mouse models, highlighting their potential role in neurodegenerative disease progression (Wu et al., 2021). A *Cell Metabolism* study in 2017, with an outbreak from 2019 to the end of 2022, examined lactate shuttling between glial cells and neurons, revealing how elevated ROS levels promote lipid synthesis in neurons and lipid droplet accumulation in glial cells via the APOE/D pathway. The study emphasized the importance of metabolic cooperation between glial cells and neurons in neurodegenerative diseases and provided new perspectives and potential therapeutic strategies for slowing down neurodegeneration by modulating lipid metabolism (Liu et al., 2017).

## 4 Discussion

The paper analyzes the evolution of research on lipid droplets in the central nervous system between 2000 and 2024 through bibliometric and visual analysis using CiteSpace and VOSviewer software. The objective is to identify the current state of research, major trends, and areas of interest, providing a foundation for future studies. Data was collected from the Web of Science database, identifying 1,066 relevant publications. The analysis showed a steady increase in studies over time, with acceleration in recent years, particularly after 2018. The countries and institutions that contributed the most are the United States, China, Germany, Italy, and India, with research centers such as Johns Hopkins University and the University of Cambridge among the key players. Miguel Lopez was identified as the researcher with the highest number of publications. Current research focuses on the role of lipid droplets in processes such as oxidative stress, neurodegenerative inflammation, and diseases like Alzheimer's and Parkinson's. Lipid droplets, which are usually rare in the nervous system, tend to accumulate under pathological conditions, especially in glial cells, altering metabolism and contributing to inflammatory processes. Studies on animal models have shown that their accumulation can precede neurodegeneration and that their regulation could be a potential therapeutic target. The analysis of keywords and citations has made it possible to identify the most relevant research topics and their temporal evolution. A growing interest has been observed in energy metabolism, the role of lipid droplets in immune response regulation, and their impact on the blood-brain barrier. Moreover, recent studies have highlighted the link between diet, obesity, and alterations in lipid droplets, suggesting that nutrition could influence the progression of neurodegenerative diseases. The paper suggests strengthening international collaboration among researchers and institutions to further investigate the physiological and pathological functions of lipid droplets in the brain. It emphasizes the importance of future research aimed at clarifying lipid droplets role in neuroinflammation and neurodegeneration, with potential implications for developing targeted therapeutic strategies.

Neuroinflammation and oxidative stress are hallmarks of neurodegenerative diseases (Ransohoff, 2016; Wilson et al., 2023; Houldsworth, 2024; Glass et al., 2010). In response to stressors like inflammation, oxidative stress, or aging, microglia in both mouse and human brains seem to preserve intracellular homeostasis within certain subpopulations. These subpopulations notably accumulate lipid droplets, generate elevated levels of ROS, and release proinflammatory cytokines, exhibiting a unique transcriptome signature driven by innate inflammation (Marschallinger et al., 2020). Under typical conditions, the body's antioxidant system regulates redox homeostasis by scavenging ROS and reactive nitrogen species (RNS). Oxidative stress occurs when oxidant production surpasses the antioxidant system's capacity, often due to factors like external injury, inflammation, and ischemia or hypoxia (Jomova et al., 2023). This leads to an accumulation of free radicals, including excessive ROS and RNS, beyond the cell's regulatory range, resulting in oxidative damage to cellular structure and function (Pisoschi and Pop, 2015). Lipid droplet formation in glial cells under oxidative stress reduces ROS levels and prevents polyunsaturated fatty acid oxidation, thus maintaining lipid homeostasis (Moulton et al., 2021). Neuroinflammation is an inflammatory activity following damage to the CNS, usually caused by injury, infection, stress, ischemia, and other stimuli that cause an increase in the permeability of the blood-brain barrier and infiltration of peripheral immune cells into the CNS (Ryan et al., 2022). Subsequently, glial cell activation leads to the release of inflammatory mediators like cytokines and chemokines. Persistent or excessive neuroinflammation results in neuronal damage and degeneration, impairing neurological function and potentially causing neurological disorders (Pintado et al., 2012; Corwin et al., 2018). Lipid droplet accumulation in neurons and glial cells may trigger an inflammatory response and is considered an early indicator of neurodegenerative changes. For example, in stroke models, increased lipid droplet accumulation in microglia influences pro-inflammatory phenotypic changes, leading to elevated secretion of factors like TNF-α, IL-6, and IL-1 (Kwon et al., 2017; Okada et al., 2022), which exacerbate neuroinflammation. LPS-induced accumulation of intracellular lipid droplets in microglial cells may increase the expression of the lipid droplet-associated protein PLIN2 (perilipin-2) through activation of the p38i/β and PI3K/Akt pathways to promote lipid droplet production (Khatchadourian et al., 2012).

Over a century ago, Alois Albert's initial report on Alzheimer's disease identified unusual fat deposits in the brain of the first diagnosed patient (Möller and Graeber, 1998; Alzheimer, 1907; Alzheimer et al., 1995). Since then, however, research on the disease has focused on Aβ and Tau proteins (Busche and Hyman, 2020), ignoring the important aspect of abnormal lipid metabolism. Indeed, the pathological accumulation of lipid droplets precedes the formation of Aβ and neurogenic fiber tangles, which may serve as an early pathological marker of Alzheimer's disease. Recent research has concentrated on the 'lipid pathology' of neurodegenerative diseases, particularly Alzheimer's disease (Li Y. et al., 2024), with ongoing inquiries into the mechanisms underlying glial-lipid pathology in Alzheimer's (Van Den Brink et al., 2018). A study in *Nature* highlights that APOE, a lipid-associated risk allele for Alzheimer's, is significantly upregulated in patient microglia. Additionally, microglia derived from human induced pluripotent stem cells with APOE risk variants exhibit increased lipid droplets (Haney et al., 2024). The study published in *Cell Metabolism* by the University of California, San Diego utilizes *in situ* imaging to reveal the pathological process by which lipid transfer between brain cells in Alzheimer's disease leads to the accumulation of lipid droplets in microglia. In the early stage of tauopathy, neuronal AMPK deficiency induces excessive accumulation of lipid droplets in microglia, which in turn triggers pro-inflammatory phenotypic transformation, oxidative stress and phagocytosis dysfunction, creating a vicious cycle of neurodegeneration and inflammation (Li J. et al., 2024).

Based on the research hotspots revealed by bibliometrics, it can be seen that there are still some gaps in the field of lipid droplets in the nervous system, which can be further explored in the following directions in the future: firstly, in terms of mechanism, the existing results are mostly based on the Drosophila and rodent models, and there is a lack of human-specific mechanisms to validate the metabolism of lipid droplets in non-human primate or organoid models. Secondly, the mechanism of lipid droplet dynamic regulation is still not completely clear, and it is necessary to track the spatial and temporal distribution of lipid droplets during the dynamic process by combining with *in vivo* imaging technology. In addition, most of the current studies on lipid droplets focus on the role of lipid droplets in neurons, microglia, and astrocytes, and less on lipid droplets in oligodendrocytes, and the heterogeneity of lipid droplet functions in different subtypes of glial cells needs to be further analyzed. From a clinical translational perspective, the screening of lipid droplet-related biomarkers and their potential application in the early diagnosis of the disease are worth exploring, and changes in the markers may reflect the stage of the disease, which may help to track the progression of the disease or become a therapeutic target. The much-anticipated PLIN family, key functional proteins on the surface of lipid droplets, can regulate the dynamic balance of lipid droplets involved in lipid storage, catabolism, and the maintenance of metabolic homeostasis, but the mechanism of their spatiotemporal-specific expression in the CNS is not clear.

With the rapid development of systems biology and artificial intelligence technology, the application of modeling technology has gradually highlighted its advantages in mechanism analysis and clinical translation, and interdisciplinary integration may become an important paradigm to break through the limitations of traditional experimental research. Based on recent findings, the Hammerstein model may provide an effective quantitative modeling approach to investigate the triggers of lipid droplet accumulation in the central system (e.g., oxidative stress and neuroinflammation).The Hammerstein model can simulate the threshold regulation and saturation effects of LDs in glial cells/neurons through the nonlinear module, and the linear part can describe the memory effects of inflammatory responses and metabolic signaling, metabolic signaling and other dynamic processes with memory effects. It is worth noting that the nonlinear module can be parametrically characterized based on models of neural networks or stochastic dynamical systems when there are multifactorial stochastic interferences in the interactions between lipid droplets and the CNS (Burrascano et al., 2024; Angiulli et al., 2024).

This pioneering bibliometric analysis of lipid droplet research visualizes the importance of lipid droplets in the brain, focusing on current status and research hotspots, despite certain limitations. Due to the limitations of some objective factors, such as Web of Science can provide data standardization and format compatibility, which can be directly recognized by the mainstream bibliometric software VOSviewer and CiteSpace; while the export formats of other databases such as PubMed and Embase may lack key fields such as reference lists, institutional information, or field naming styles that are incompatible with the software. In addition, considering that the documents in the WoS core set have been carefully selected and complete citation network data (e.g., the number of citations and reference list of each document) are provided, which is crucial for co-citation analysis, author collaboration networks, and other studies. Therefore, we visualized and analyzed the literature entries extracted from the WoS database, but there is still a possibility that literature from other databases only, such as PubMed and Embase, were excluded from this study (Guo et al., 2022). In the future, we hope that we can learn and enhance the technical tools such as specific programming to realize the effective integration and cross-database analysis of English databases. Secondly, considering that errors in the processing of specialized terminology by translation tools may introduce new biases, and that SCI/SSCI journals published in English occupy the majority of the journals, and non-English results are also mostly entered into the retrieval system in the form of English abstracts, our study included only English-language literature to ensure the controllable quality of the study, and there is a possibility that non-English-language literature on related topics may have been omitted.

## 5 Conclusion

Over the past 20 years or so, the precise role of lipid droplets in aging and neurodegenerative diseases, including neuroinflammation, neuronal oxidative stress, and their accumulation, remains unclear. Understanding these mechanisms could aid in identifying therapeutic targets and clarifying clinical interventions for lipid droplet metabolism disorders in neurological conditions (Zhang et al., 2025). The rising number of publications in this research area reflects its increasing interest and recognition. We recommend enhanced international collaboration among countries, institutions, and researchers to advance fundamental research and explore clinical applications of lipid droplets. Future research should focus on clinical evaluation and innovative methodologies for studying lipid droplets. We hope that this bibliometric analysis will provide useful information for the future. The rising number of publications in this research area reflects its increasing interest and recognition. We recommend enhanced international collaboration among countries, institutions, and researchers to advance fundamental research and explore clinical applications of lipid droplets. Future research should focus on clinical evaluation and innovative methodologies for studying lipid droplets.

## Data Availability

The original contributions presented in the study are included in the article/supplementary material, further inquiries can be directed to the corresponding author.
